# Total laparoscopic hysterectomy (TLH) with endosuturing compared with conventional technique using energy sources

**DOI:** 10.52054/FVVO.13.2.018

**Published:** 2021-06-28

**Authors:** V Marwah, S Dutta, S Kedia, P Mittal

**Affiliations:** Division of Minimally Invasive Gynecology Surgery, Institute of Minimal Access, Metabolic & Bariatric Surgery, Max Healthcare Institute Ltd., Saket, New Delhi, India.

**Keywords:** Total laparoscopic hysterectomy (TLH), endosuturing, energy sources, thermal injuries

## Abstract

**Background:**

The aim of the study was to demonstrate the technique of total laparoscopic hysterectomy (TLH) with intra-corporeal endosuturing using simple sutures and basic surgical instruments and compare with TLH using electric coagulation equipment i.e. energy sources with regard to operative time, blood loss, postoperative stay and pain scores.

**Methods:**

A retrospective study was undertaken, in Max Super Specialty Hospital Saket, from June 2015 to May 2018, which included 586 cases of TLH (for benign gynecological conditions), of which 287 were performed using intra-corporeal endosuturing (Group 1) and 299 were performed using energy sources (Group 2). To avoid bias, baseline matching was done for body mass index (BMI), indications for surgery, size of uterus, previous abdominal surgeries and comorbidities like diabetes and hypertension after which there were 172 patients in each group.

**Results:**

The mean age of patients was 48.24 ± 6.76 years. All operative outcomes including operative time (104.1 ± 22.6 vs 107.6 ± 32.6 mins, p=0.25), blood loss (78.9 ± 101.6 vs 99.7 ± 177.6 ml, p=0.19), pain score (2.5 ± 1.3 vs 2.7 ± 1.2, p=0.13) and post-operative stay (2.05 ± 0.2 vs 2.07 ± 0.3 days, p=0.36) were similar between the two groups. Uterine size was the major determinant of operative time and operative blood loss.

**Conclusion:**

TLH with intracorporeal endosuturing can be performed safely and gives results comparable with TLH performed using energy sources. Advancement in suturing devices can decrease operative time further and potentially make it easier and more acceptable.

## Introduction

Advances in medical technology and high resolution camera imaging has led to minimally invasive gynecologic surgery becoming the gold standard for benign conditions (Han and Advincula, 2019), (Bhagavath and Benjamin, 2015). Hysterectomy is the commonest gynaecological procedure, and over the years, techniques have evolved from abdominal to laparoscopic assisted vaginal hysterectomy to the contemporary total laparoscopic hysterectomy (TLH), first introduced in 1989 by Reich et al (1989).

TLH has demonstrated advantages over abdominal hysterectomy including shorter hospital stay, lower infection rates, better cosmetic effect with less pain and lower post-operative adhesion rates (Reich et al., 1989). Before advanced energy sources were available, laparoscopic surgical procedures used sutures and basic instruments, pioneered by Kurt Semm, who initiated intra and extra-corporeal knotting to achieve endoscopic haemostasis (Litynski, 1998). Advances in electrosurgical and vessel sealing devices along with introduction of various uterine manipulators simplified laparoscopic surgery further, thus making TLH more widely acceptable (Pandey et al., 2014).

Since the advent of TLH, energy sources have evolved from monopolar electro-surgery, bipolar energy to ultrasonic techniques which may decrease surgical morbidity due to vascular and visceral injuries (Pandey et al., 2014). Conventional bipolar electrocautery and ultrasonic scalpels are commonly used especially in the early phase of the learning curve of hysterectomy. Despite rapidly improving technical equipment and surgical skills, the rate of electrosurgical complications during energy transmission has been reported to be as high as 25.6% which is second only to the most common complication of injury due to Veress needle or primary trocar entry (Huang et al., 2014a).

TLH with endosuturing emphasises the technique of TLH using intracorporeal endosuturing with no use of additional energy sources for dissection or cutting. The surgical procedure is completed with the help of basic instruments and sutures following the avascular planes for surgical navigation (Agarwal et al., 2010; Cho et al., 2007). The present study demonstrates this technique and has evaluated its benefits and constraints, in comparison with the conventional technique using energised dissection.

## Methods

### Patients and setting

Consecutive patients with benign gynecological conditions, who underwent total laparoscopic hysterectomy, under the Division of Minimally Invasive Gynecology, Max Super Specialty Hospital, Saket, New Delhi, India from June 2015 to May 2018 were included in the study. Inclusion criteria included all benign uterine diseases. All surgery were operated by the same surgeon. Max Super Specialty Hospital is a tertiary care centre with JCI accreditation located in North India serving to international and national referrals. Ethical approval was obtained from the institute review board. The study was a retrospective analysis.

### Preoperative evaluation

Patients’ baseline characteristics were analysed including age, symptoms, past medical and previous surgical history and all patients underwent routine investigations including complete blood count (CBC) and biochemistry tests along with a pre- anesthetic check-up. After patient counseling, including information about the procedure, complications and post-operative course, informed consent was taken.

### Intervention and follow up

The hospital’s centralised patient record system maintains the record of patients’ clinical data including demographics, procedural details, operative findings, complications, postoperative parameters including operative time, blood loss, visual analogue scale (VAS) score for pain and post- operative stay.

586 patients underwent TLH of which endosuturing was used for 287 cases (Group 1) and energy sources were used for 299 cases (Group 2). The patients were discharged from the hospital within 36-48 hours except for patients with significant co-morbidities. The patients were closely monitored post-operatively for any evidence hemorrhage, or other surgical complications.

All patients were followed up at one and six weeks after surgery.

### Statistical analysis

The baseline features that could have affected the outcomes were different between group 1 and group 2 of the entire cohort (Table I). Hence, to avoid bias, baseline matching was done for BMI (<35 and >=35), indications for surgery, size of uterus (<16 and >=16), previous abdominal surgeries and comorbidities such as diabetes and hypertension (all of these, either yes or no). This left 172 patients in each group.

**Table I:** Comparison of baseline disease characteristics between the two methods of surgery before matching.

Features	All patients (n=586)	TLH without energy source (n=287)	TLH with energy source (n=299)	P value
Age (mean + SD^1^)	49.1 + 6.0	48.9 + 6.3	49.2 + 5.8	0.54
BMI^2^ (mean + SD)	28.9 + 5.3	28.3 + 4.7	29.5 + 5.7	0.01
Indications for surgery, n (%)*				
Myoma	391 (66.7)	189 (65.9)	202 (67.6)	0.66
Adenomyosis	221 (37.7)	122 (42.5)	99 (33.1)	0.02
Ovarian cyst	31 (5.3)	11 (3.8)	20 (6.7)	0.12
Post-menopausal bleeding	53 (9.0)	28 (9.8)	25 (8.4)	0.56
Endometriosis	116 (19.8)	51 (17.8)	65 (21.7)	0.23
DUB^3^	10 (1.7)	4 (1.4)	6 (2.0)	0.75
Co-morbid conditions n (%)				
Hypertension	154 (26.3)	78 (27.2)	76 (25.4)	0.63
Diabetes	53 (9.0)	23 (8.0)	30 (10.0)	0.39
Previous abdominal surgeries, n (%)	317 (54.1)	136 (47.4)	181 (60.5)	0.001
LSCS^4^	223 (38.1)	90 (31.4)	133 (44.5)	0.001
Laparoscopic surgeries	130 (22.2)	65 (22.6)	65 (21.7)	0.79
Open surgeries	54 (9.2)	18 (6.3)	36 (12.0)	0.02
Grades of endometriosis n (%)				
Mild	52 (8.9)	21 (7.3)	31 (10.4)	0.42
Moderate	16 (2.7)	7 (2.4)	9 (3.0)	
Severe	54 (9.2)	25 (8.7)	29 (9.7)	
Mean + SD, size of uterus (weeks)	12.4 ± 5.0	11.7 ± 4.3	13.1 ± 5.5	0.001

*there can be more than one indication for surgery.

^1^ standard deviation; ^2^ body mass index; ^3^ dysfunctional uterine bleeding; ^4^ Lower segment caesarean section.

The groups were compared using Student t-test for quantitative measurements and by chi-square test for qualitative variables. P values < 0.05 were considered statistically significant. All statistical analyses were performed using SPSS version 20.

### Technique

**Figure 1:**
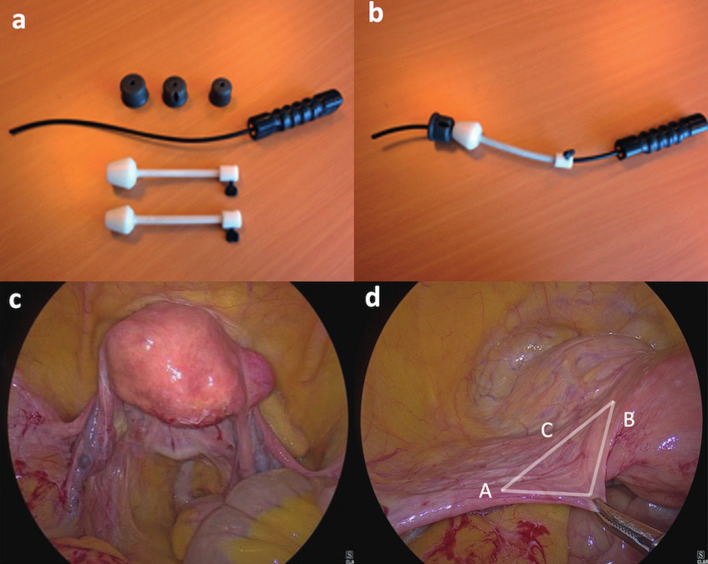
— (a,b) Uterine manipulator with culdotomy cups a) disassembled, b) assembled; c) Intraoperative view of Uterus in anteverted position with the help of uterine manipulator; d) Intra-operative demonstration of laparoscopic dissection of Avascular Triangle (A-round ligament; B-uterus; C-uterovesical fold of peritoneum) for skeletonization of the uterine bundle.

The patient was placed in dorso-lithotomy with legs positioned at 30° flexion, placed in stirrups and sterile drapes applied. An indwelling foley urethral catheter was placed. Our self-designed uterine manipulator was then inserted for better mobilisation of uterus, visibility of the vault and for the safety of bladder and ureters (Figure 1a, b).

Once a pneumoperitoneum was created, the primary trocar of 10 mm was inserted through the umbilicus or supraumbilical port (2-3 cm above the umbilicus) depending on the size of the uterus and previous history of abdominal surgeries. If required a second 10 mm port was placed in the mid clavicular line in subcostal region depending on size of uterus and need for morcellation. 2 or 3 secondary 5mm ports were placed under direct vision depending upon the size of uterus and pathology involved.

Visceral and omental adhesions when present (especially with a history of previous surgery) were dealt with by blunt and sharp dissection along avascular planes. Adhesions were divided after identifying for transparency of tissues on the edge of the Metzenbaum scissors after traction on the viscera and omentum with atraumatic graspers.

**Figure 2:**
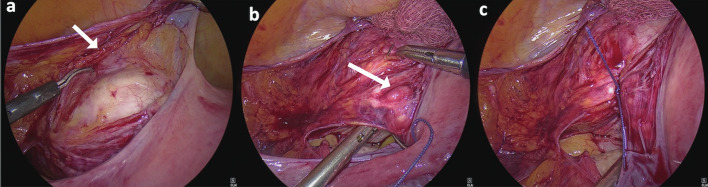
— Intra-operative demonstration of a) Dissection of utero-vesical fold of peritoneum for skeletonization of uterine bundle; b) Intra- corporeal suturing of the uterine bundle (white arrow) while taking the stump; c) Completion of suturing of left uterine artery and veins.

Pelvic structures were identified including the ureters (Figure 1c). The uterus was anteverted and deviated towards the right side by the assistant at the vaginal end. Approach of uterine vessels first was adopted. Advantage was taken of the ‘Avascular Triangle’ bounded laterally by the round ligament, medially by uterus and inferiorly by the uterovesical fold of peritoneum with the uterine artery and vein running in the base of the triangle above the indentation of the culdotomy cup (Figure 1d). A small nick was given just above the uterovesical fold of peritoneum, 3-4 cm lateral to the uterus in the anterior leaf of broad ligament. The nick was extended medially over the uterine vessels and superiorly up to the round ligament. The posterior leaf was opened in a similar manner and a window created in the avascular triangle for skeletonization of uterine bundle (composed of uterine artery and veins). The uterovesical fold of peritoneum was then opened and dissection done over and against the culdotomy cup (over the uterine manipulator) shifting the urinary bladder inferiorly and displacing the ureter laterally and inferiorly where it is much less susceptible to injury (Figure 2a). This further delineated the uterine bundle for placement of suture (Figure 2b). Left uterine bundle was ligated intracorporeally using No.1-0 Polyglactin 910 suture (Figure 2c). The procedure was then repeated on the right side. Blanching of the uterus confirmed successful ligation of uterine arteries on both sides.

Following this, the left fallopian tube and utero- ovarian ligament were ligated with transfixation sutures. Then the left round ligament was ligated in the similar manner. Polyglactin 910 No. 1-0 suture was used to ligate all the stumps intracorporeally. Round and utero-ovarian ligament were ligated separately as lesser tissue in one stump ensures better haemostasis. In cases where salpingo-oophorectomy was carried out, the infundibulopelvic ligament was ligated instead of fallopian tube and ovarian ligament, after visualising the ureter. (Figure 3a ,b). The procedure was then repeated on the right side.

**Figure 3:**
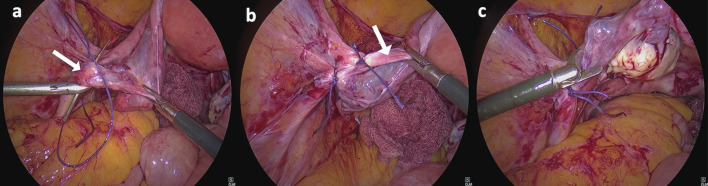
— Intra-operative demonstration of a) Laparoscopic intra-corporeal suturing of the infundibulopelvic (white arrow) ligament; b) Complete suturing of infundibulopelvic and round ligament (white arrow); c) Cutting of adnexal stumps via hooked scissors on left side.

The uterus was retroverted and bladder dissection was completed anteriorly.

Subsequently the uterus was then anteverted and hooked scissors were used to cut the bilateral adnexal pedicles (Figure 3c) following which the uterine pedicles and cardinal ligaments were also cut on both sides.

During this step, firm pressure was maintained on the vagina with the culdotomy cup of the manipulator from vaginal end. This not only helps in dissection but also ensures lesser blood loss from the vaginal vault due to its pressure effect on the capillaries. Culdotomy was completed using hooked scissors to thin out and make small perforations along the vaginal vault all around (Figure 4a). This was done to prevent complete opening of the vault from one side which would bring the culdotomy cup inside and alter the circular plane of dissection.

**Figure 3:**
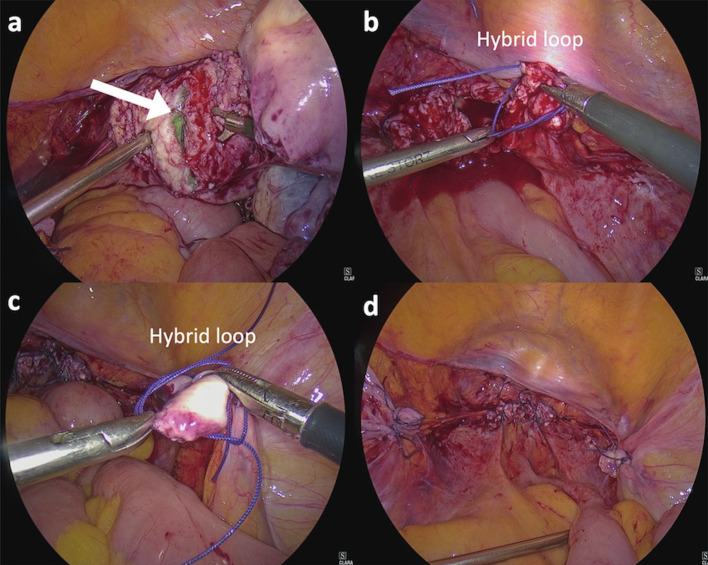
— Intra-operative demonstration of a) Laparoscopic cutting of cardinal ligament and culdotomy in progress using hooked scissors on left side (white arrow indicates small perforations along the vaginal vault); b) Application of hybrid suture loop over the right uterine pedicle; c) Application of hybrid suture loop over the right infundibulopelvic ligament; d) Vaginal vault and pedicles after the procedure was completed.

The uterine manipulator was then removed leaving behind the culdotomy cup which was removed later. The uterus was then detached from the vault using hooked scissors.

Depending upon the size of uteri, extraction was performed vaginally in most cases. In case of narrow introitus or big size of the uterus, morcellation was done by converting the upper left 10 mm port into 15 mm port. Culdotomy cup was removed along with uterus vaginally. For anchoring and closure of the vault; figure-of-8 sutures were taken on both the angles taking the posterior vaginal wall, uterosacral ligament, cardinal ligament and the anterior vaginal wall with third figure-of-8 suture in the middle of the vault to complete the closure. Polyglactin 910 No 1 suture was used for closure of vault.

To ensure complete hemostasis, all pedicles were further secured with ‘Hybrid Suture Loops’ which are pre-formed extracorporeal loops (with Polyglactin 910 No 1-0), tied intracorporeally (Figure 4 b, c).

Sometimes small active blood vessels on the bladder and peritoneal surfaces were observed. For these we tried compression with simple X-ray highlighted gauze pieces which proved to be effective especially for small bleeders on the bladder surface as the oozing usually took 3-4 minutes to stop in accordance with the coagulative profile of patient. After this the laparoscope was withdrawn and patient placed in supine position and intra- abdominal pressure was released. This way we could also buy some time to observe for the small capillary bleeds if present. After 5 minutes, again pneumoperitoneum was created and all stumps were visualised (Figure 4d) and small bleeders (if any) visualised. If oozing still persisted, we used the polyglactin no 3-0 to ligate the same which was rarely required. Abdominal lavage was done and hemostasis ensured. To rule out ureteric injury; both the ureters were identified and their peristalsis was observed routinely and in cases of previous caesarean surgery, methylene blue dye diluted in normal saline was filled in a retrograde manner into the bladder to check for injuries. 10mm or bigger port sites were closed with a port closure needle. Skin closure was with staples.

## Results

A total of 586 patients underwent TLH between June 2015 and May 2018. Of these, 287 were operated without energy source (Group 1) and 299 were operated using energy source (Group 2) (Table I). After baseline matching, there were 172 patients in each group.

## Comparison of baseline demographic features, indications for surgery, and disease characteristics between two methods

Because of matching, baseline parameters like age, BMI, indications for surgery, H/O previous abdominal surgery, grades of endometriosis, and mean size of uterus were comparable between the two groups (Table II). The most common indication for surgery was myoma uterus followed by adenomyosis (Table II).

**Table II:** Comparison of baseline disease characteristics between the two methods of surgery after matching.

Features	TLH without energy source (n=172)	TLH with energy source (n=172)	P value
Age (mean + SD^1^)	48.2 + 6.7	48.6 + 6.0	0.56
BMI^2^ (mean + SD)	28.0 + 4.5	28.4 + 5.0	0.47
Indications for surgery, n (%)*			
Myoma	120 (69.8)	120 (69.8)	1.00
Adenomyosis	60 (34.9)	60 (34.9)	1.00
Post-menopausal bleeding	13 (7.6)	13 (7.6)	1.00
Endometriosis	33 (19.2)	33 (19.2)	1.00
Ovarian cyst	7 (4.1)	10 (5.8)	0.46
DUB^3^	2 (1.2)	4 (2.3)	0.41
Co-morbid conditions n (%)			
Hypertension	38 (22.09)	38 (22.09)	1.00
Diabetes	5 (2.9)	5 (2.9)	1.00
**Previous abdominal surgeries, n (%)**	102 (59.3)	102 (59.3)	1.00
LSCS^4^	68 (39.5)	77 (44.8)	0.33
Laparoscopic surgeries	47 (27.3)	35 (20.3)	0.79
Open surgeries	15 (8.7)	22 (12.8)	0.22
Grades of endometriosis n (%)			
Mild	13 (7.6)	17 (9.9)	0.62
Moderate	4 (2.3)	5 (2.9)	
Severe	17 (9.9)	17 (9.9)	
Mean + SD, size of uterus (weeks)	12.1 ± 4.6	12.6 ± 5.2	0.29

*there can be more than one indication for surgery.

^1^ standard deviation; ^2^ body mass index; ^3^ dysfunctional uterine bleeding; ^4^ Lower segment caesarean section.

## Comparison of post-operative outcomes between the two methods of surgery

The post-operative outcomes including operative time, blood loss, pain score after surgery, and mean post-operative stay was comparable between the two groups (Table III). The only significant predictor of blood loss and duration of surgery was uterine size, and both outcomes were greater in patients with uterine size of uterus greater than 16 weeks (Table IV). Blood transfusion was required for 2 patients in Group 1 and 3 patients in Group 2. Overall complications included urinary tract injuries in which 2 cases of bladder injury were reported in group 2. There were no cases of vaginal stump infection or hematoma, secondary hemorrhage, bowel injury or abdominal wound complication in both the groups.

**Table III:** Comparison of intra-operative findings and outcomes between the two methods of surgery.

Features	TLH without energy source (n=172)	TLH with energy source (n=172)	P value
Operative time — Mean + SD^1^	104.1 ± 22.6	107.6 ± 32.6	0.25
Blood loss (Median, IQR^2^)	78.9 ± 101.6 25 (25 – 100)	99.2 ± 177.6 50 (25 – 100)	0.19
Pain score after surgery (VAS 3 – scale of 0-10)	2.5 ± 1.3	2.7 ± 1.2	0.13
Mean post-operative stay after surgery (days)	2.05 ± 0.2	2.07 ± 0.3	0.36

*there can be more than one indication for surgery.

^1^ Standard Deviation; ^2^ Inter quartile range; ^3^ Visual analogue scale.

**Table IV:** Analysis for possible predictors of operative time and blood loss.

	Variables	Operative time	P value	Blood loss	P value
BMI^1^ (kg/m^2^)	<35 (n=318)	105.1 + 27.8	0.79	91.0 ± 148.6	0.25
	>35 (n=26)	107.3 ± 31.4		65.4 ± 84.6	
Size uterus (weeks)	<16 (n=272)	100.0 ± 21.5	<0.001	71.2 ± 80.6	0.009
	>16 (n=72)	120.2 ± 37.5		156.8 ± 265.8	
Type of surgery	Energy-less (n=172)	104.1 ± 22.6	0.25	78.9 ± 101.6	0.19
	With energy sources (n=172)	107.6 ± 32.6		99.2 ± 177.7	
Previous surgery	Absent (n=140)	102.4 ± 27.1	0.052	86.1 ± 95.9	0.75
	Present (n=204)	108.3 ± 28.6		91.2 ± 170.1	

^1^ Body mass index

## Discussion

TLH has gained increasing popularity following the pioneering work of Reich and colleagues (1989). The use of energy sources has improved haemostatic techniques and contributed to the rapid assimilation of the laparoscopic approach in gynaecology. These advances have diminished the possible disadvantages for some surgeons of laparoscopic surgery such as limited peritoneal access, long surgical instrument length, two dimensional imaging and little tactile feedback (Gözen et al., 2007; Agarwal et al., 2007; Gallagher et al., 1998; Hanna et al., 1998).

These advantages have simplified key-hole surgeries to an extent, but energy devices may also contribute to surgical complications. They may be associated with inadvertent thermal and mechanical injuries due to direct application, stray currents, capacitive coupling, direct coupling, and alternate site burns (Huang et al., 2014a; Guzman et al., 2019; Steinemann et al., 2016; Odell, 2013).

Gross assessment of injuries associated with energy sources as studied by Tulikangas et al (2001) revealed that average length of injury for bipolar cautery was 0.4±0.2 cm on the ureter, 1.3±0.2 cm for the bladder, and 1.3±0.2 cm for the rectum. Thus, TLH using energy sources contribute to lateral thermal spread which is eliminated when using intracorporeal endosuturing thus potentially making it a viable and safer alternative for the patient.

Electro-surgical energy in gynaecological surgery may lead to aerosol generation with potential exposure of healthcare workers to bacteria and viruses with risk of SARS-Cov-2 being no exception (European Society for Gynaecological Endoscopy, 2020; Mallick et al., 2020). Reducing energy usage may also be one partial solution to environmental challenges. The impact on the environment due to the disposable materials produced by the use of single-use energy sources also needs evaluation by the healthcare industry (Thiel et al., 2015).

Treatment of complications induced because of thermal injury are associated with morbidity far more than complications induced because of endosuturing. Moreover, injury to visceral organs caused during endosuturing does not devascularise the site of injury and restoration of normal functioning is more rapid. Furthermore, culdotomy performed by hooked scissors does not devascularise the vault reducing the chance of vault dehiscence and improved healing. Furthermore the symptoms of bowel perforation due to thermal injury usually appear later (4-10 days) than those due to traumatic perforation (usually 12-36 hrs) (Huang et al., 2014b). There are certain cases where use of electrosurgery needs extreme care and caution as in patients with pace makers and implantable cardio version devices (García Bracamonte et al., 2013). In such patients TLH with intracorporeal endosuturing may be a safer option.

To emphasise the judicious use of energy sources, Agrawal et al. (2010) demonstrated laparoscopic cholecystectomy without energised dissection taking advantage of the avascular holy planes. The use of endosuturing techniques in gynaecology were described way back in 1996 by Ostrzenski (Ostrzenski et al., 1996; Ostrzenski et al., 1998), where all the pedicles were secured with extracorporeal sliding and intracorporeal two- turn flat square knot technique, but the dissection and cutting were accomplished with monopolar electrocoagulation. Further demonstration of the use of energy less dissection was made by Rotithor et al. (2015) during colpotomy, to reduce the incidence of vaginal cuff dehiscence and injury to the bladder and ureter. Recently, Hye Won Kang et al. (2016) in a retrospective analysis of 746 patients, described the suturing techniques for performing TLH with extracorporeal knots and the use of monopolar cautery for culdotomy, with a conclusion that ‘classic’ suturing technique had tolerable complications and blood loss.

Therefore, there has been a constant endeavor to perform TLH with the same suturing techniques as are done in open abdominal hysterectomy. The present study has tried to bridge this gap by describing an intracorporeal endosuturing technique using conventional laparoscopy instruments and endosutures following the avascular surgical planes. Our technique is different from the previous techniques as the ligation of uterine vascular bundle, and round and tubo-ovarian / infundibulopelvic ligaments were performed with intracorporeal endosuturing and culdotomy was done over the cup with the help of hooked scissors without the use of monopolar cautery i.e. energy sources was not used in the complete procedure. Moreover, the present study has also compared the operative outcomes between two techniques of performing TLH: with intracorporeal endosuturing without energised dissection and TLH with energised dissection. The uterine arteries were ligated first (uterine first approach) to decrease the perfusion pressure and uterine blood volume thereby reducing the blood loss during further dissection (Kale et al., 2015). Furthermore, the absence of smoke in the classic suturing method helped in maintaining clear endovision, which facilitated enhanced delineation and identification of vital structures. The absence of smoke reduced the need of repeated cleaning of the lens and the use of smoke extractors thereby decreasing the amount of carbon dioxide used contributing in the efforts of decreasing the carbon footprint (Power et al., 2012; Gilliam et al., 2008). The post-operative outcomes including blood loss, operative time, pain score and post-operative stay were also comparable between the two techniques. Both the patients in whom there was a bladder injury had extensive utero-vesical adhesions because of previous multiple caesarean sections, and underlying endometriosis. The most important determinant of intra-operative blood loss and operative time was the size of uterus.

This study is one of the first of its kind which has compared the operative outcomes between the time-tested technique of energised dissection, and an intracorporeal endosuturing technique that still remains relatively unexplored. The study had a reasonably large cohort size to strengthen the results.

Though, the baseline characteristics such as BMI, size of uterus, indications for surgery, and history of prior abdominal surgery were different between the two groups (due to retrospective design and lack of randomization), we could overcome these limitations by matching for these variables. The technique does require the surgeon to have good laparoscopic suturing skills which can cause the outcomes to vary and possibly has a slow learning curve which we believe, can be developed with practice.

To conclude, TLH with intracorporeal endosuturing is a safe, environmentally friendly, economic and a viable option. In the future, we need to improve on the endosuturing devices which will compensate for the need of endosuturing skill, making it more appreciable and acceptable amongst budding surgeons.
